# Identification of acid reflux cough using serial assays of exhaled breath condensate pH

**DOI:** 10.1186/1745-9974-2-3

**Published:** 2006-04-11

**Authors:** John Hunt, Yuanlin Yu, James Burns, Benjamin Gaston, Lina Ngamtrakulpanit, Dorothy Bunyan, Brian K Walsh, Alison Smith, Stephanie Hom

**Affiliations:** 1 Division of Pediatric Respiratory Medicine, Box 800386, University of Virginia, Charlottesville, Virginia 22908, USA; 2 Center for Laryngeal Surgery and Voice Rehabilitation, Massachusetts General Hospital, One Bowdoin Square, Boston, MA 02114, USA

## Abstract

**Background:**

Chronic cough is a common problem, frequently caused or exacerbated by acid reflux. Diagnosis of acid reflux cough is haphazard currently, often relying on long therapeutic trials of expensive medications. We tested the hypothesis that the most relevant mechanistic component of acid reflux in chronic cough is when it rises to the level of the airway where acid can potentially be aspirated. We further wished to determine if multi-sample exhaled breath condensate (EBC) pH profiles can identify chronic cough patients likely to respond to proton pump inhibitor therapy.

**Methods:**

59 subjects were recruited for this study. Initially we examined EBC pH (gas-standardized with Argon) in the setting of 15 experimental pharyngeal acid challenges to determine duration of EBC acidification. Subsequently, we enrolled 22 healthy subjects to determine a normal multi-sample exhaled breath condensate pH profile over 1–3 days. We additionally obtained multi-sample EBC pH profiles in 22 patients with chronic cough. These samples were timed to occur after coughing episodes. Exhaled breath condensate pH was measured after gas standardization.

**Results:**

We found that exhaled breath condensate pH is substantially reduced for approximately 15 minutes after pharyngeal acid load. Healthy subjects rarely have any low EBC pH values (defined as < 7.4 based on a normative reference range from 404 healthy subjects). Patients with chronic cough who subsequently responded well to proton pump inhibition (n = 8) invariably had one or more cough episodes associated with EBC acidification. No patient who had normal EBC pH with each of their cough episodes reported a clinically relevant response to proton-pump inhibition.

**Conclusion:**

Patients whose cough responds to proton pump inhibition have transient exhaled breath condensate acidification with coughing episodes, supporting the role of airway acidification in reflux-triggered cough. Multi-sample EBC pH profiles, involving samples collected immediately subsequent to a coughing episode, may be useful appropriately to direct therapy to those patients with cough who have relevant acid reflux.

## Introduction

Cough is a leading reason patients consult respiratory physicians. Gastric acid reflux up the esophagus is a well-recognized cause of chronic cough both in the presence and absence of underlying lung or airway diseases. Two mechanisms of this cough have been demonstrated: 1) reflux high into the laryngeal/hypopharyngeal region with laryngeal acid contact with or without aspiration into the airway; 2) esophageal acid contact. Both of these sites of acid exposure lead to cough through vagal-mediated reflex pathways and neurogenic inflammation, but importantly the first also leads to the diverse pathologies resulting from the direct acid injury to the airway[1]. In this project we tested the hypothesis that acid reflux to the level of the airway is a critical component for the triggering of cough in acid reflux cough.

Data are mixed about the utility of proton pump inhibition (PPI) for the treatment of suspected acid reflux cough[2,3], and in the United States no PPI is approved by the government for marketing and sale for this purpose. Yet, respiratory medicine physicians and otolaryngologists prescribe PPI's frequently, and with some confidence that they are effective for respiratory manifestations of acid reflux. We have been curious as to why there is a discrepancy between the equivocal efficacy of these medications in certain published studies and the evident utility of these medications in actual practice.

One explanation is that studies have enrolled the wrong patients. Most studies of acid reflux cough were designed to recruit subjects with respiratory symptoms who also had symptomatic or esophageal pH probe evidence of gastro-esophageal reflux disease (GERD). However, the amount of acid in the airway necessary to trigger airway symptoms such as cough is substantially lower than the amount of acid reflux necessary to trigger esophageal symptoms. Whereas 4% of more esophageal acid contact time may be abnormal from an esophageal standpoint, any acid contact time in the airway, even for moments, is likely capable of causing pronounced symptoms. Esophageal symptoms are commonly not present in patients with acid reflux cough[4]. And GERD symptoms are common in patients with asthma and COPD, but may not be relevant in a given patient[3]. For these reasons, enrolling subjects with GERD when studying the therapeutic efficacy of acid blockade may not be the optimal strategy, and this design flaw may explain why such studies commonly report marginal or conflicting results.

We hypothesized that acidification of the hypopharynx, such as occurs when gastric acid refluxes above the upper esophageal sphincter into the hypopharynx, should cause exhaled breath condensate(EBC) to be acidic(after gas-standardization). We examined this hypothesis by means of pharyngeal acid challenges. We then tested for spontaneous hypopharyngeal gastric acid reflux by performing EBC pH testing in patients suspected of having acid reflux cough based on history and physical examination. Over a period of one or more days, we tested for acidic breath multiple times per subject, within several minutes of coughing episodes. We compared the EBC pH profiles thus obtained with responsiveness of the chronic cough to a 1 month treatment course with twice daily PPI therapy. This comparison allowed an examination of the ability of the EBC pH profile to predict responsiveness to PPI therapy, which functions as a diagnostic gold standard of sorts. This study design also provided evidence of effectiveness of PPI therapy in the selected population.

## Methods

### Subjects

56 subjects were recruited from the region of the University of Virginia during 2005. Subjects consisted of patients with chronic cough derived primarily from the adult and pediatric pulmonology, allergy and otolaryngology clinics, as well as controls obtained by convenience within the University. The principal enrollment criterion for chronic cough patients was the intention of their doctor to initiate a therapeutic trial of proton pump inhibition as an effort to make a diagnosis of possible acid reflux cough. Chronic cough needed to have been present daily for at least 6 weeks. Minimum age was set as 5 years. To assure real-world utility of the study, subjects with chronic cough were included without regard to the presence or suspicion of other diagnoses, but solely on the basis of the physician's planned therapeutic trial. Exclusion criteria included use of PPI or H2 antagonists within the past 7 days, or a previous attempt to treat the cough with acid blockade. Additionally, if other medication regimen changes were made concurrently, the patients were excluded. No patients had undergone esophageal pH probe testing. The studies were approved by the Human Investigation Committee at the University of Virginia and all subjects provided informed consent.

### Collection of exhaled breath condensate

Individual EBC samples were collected without nose clips during 5 minutes of relaxed breathing through a single-use disposable RTube EBC collector (Respiratory Research, Inc. USA), with initial temperature of between -4 and -17°C. The RTube device consists of a polypropylene condensing surface kept chilled with a reusable aluminum cover. Two one-way valves serve to direct exhaled air appropriately through the condenser.

### Pharyngeal acid challenge

To determine how long EBC pH might stay abnormal after a pharyngeal acid challenge, 15 subjects performed EBC collection in the laboratory followed by rapid ingestion of 50 milliliters of an acidic beverage (lemonade, pH 2.8) in a reverse model of acid reflux. They then collected 6 consecutive EBC samples (5 minutes each) for 30 minutes. For comparison, 5 subjects performed the same set of EBC collections, but after ingesting 50 milliliters of tap water (pH 7.8).

### Exhaled breath condensate collection for multisample testing of EBC pH

Subjects were provided a collection kit consisting of 8 disposable RTube EBC collectors and a pre-addressed express mailing box (Single Subject Longitudinal airway pH Monitoring Kit, Respiratory Research, Inc, USA). This sampling procedure was developed specifically to create a multi-sample EBC pH profile for the subjects, which is a clear distinction from all previous published EBC studies. The subjects were asked to collect the 8 EBC samples in their home or work over a period of 1 to 4 days, to include at least 2 samples when they had not been coughing for the previous 1 hour. The remaining samples were requested to be collected specifically when the subject had experienced a coughing episode within the previous 10 minutes. Sample collection duration for each collection was requested to be 5 minutes, and no nose clips were worn. Temperature of collection was determined by the home freezer temperature (generally between -4 and -17°C), which was used to chill the aluminum that provides the cool temperature for condensation in the RTube collection system. Subjects were asked to not collect any samples within one hour of any liquid or food ingestion. After collection of each sample, the subject wrote the date and time of collection on the RTube label along with checking a box to discriminate whether this was a "cough" or "no cough/well" sample. Each sample was stored in their home freezer until 4 days had passed or all 8 collections were completed, whichever came first. At that point, the subjects placed the RTube EBC samples into the return mailing container for shipment (2-day) to the investigators' laboratory. During this shipping, samples thawed and were not temperature controlled (which had been shown in preliminary studies to not adversely affect EBC pH values). Healthy subjects provided 8 EBC samples in similar fashion as delineated above, but because they had no cough, the samples were collected at conveniently spaced times during the course of 1–4 days.

### Study protocol

After providing the multiple EBC samples as described above, chronic cough patients began taking a proton pump inhibitor as prescribed by their physician. The therapeutic regimen was determined by their doctor, and no specific medication or dosing was mandated by the study although all patients were prescribed the medication as a twice daily regimen. Assessment of response to PPI was based on a subjective scoring scale performed 1 month after starting the PPI. Subjects were asked if their cough was 0, 25, 50, 75 or 100 percent improved. 75 % or better was determined in advance of the study to be considered a "PPI responder." An improvement of 0 or 25% was considered a "non-responder." 50% improvement was considered an equivocal response.

### EBC pH assay

Upon receipt into the laboratory, data were recorded from the labels of the RTube EBC collectors and the samples removed by plunging the condensers with the internal syringe plunger. A 250 microliter aliquot of EBC was gas-standardized by bubbling with Argon for 8 minutes at 350 ml/min prior to pH measurement, which was performed with an Orion pH glass combo microelectrode attached to an Orion 520 A meter as previously reported[5]. The probe and meter had been calibrated at pH 4, 7, and 10 with standard as well as low ionic strength calibration buffers prior to each set of assays.

### Definitions of positive and negative EBC pH profiles

A positive EBC pH profile prospectively was defined as one in which one or more or the patient's coughs occurred with a concurrent low EBC pH value (equal to pH <7.4, based on the normative database previously published by Paget-Brown[6]), while at least one EBC sample collected by the patient had a normal EBC pH value. A negative EBC pH profile was defined as one in which none of the samples performed after cough had a low EBC pH value. Healthy subjects without cough were considered EBC pH profile negative by definition (because they had no cough with which a low EBC pH could occur).

### Statistical considerations

The effect on EBC pH of pharyngeal acid challenge was examined graphically and EBC pH at each time period compared by ANOVA on Ranks followed by Dunn's test. Comparison of individual EBC pH values was accomplished by Mann-Whitney Rank Sum, and the number of low EBC pH values for each group compared by Chi-squared analysis. The ability of the system of EBC collections and resulting EBC pH profile in each patient to predict cough responsiveness to PPI therapy was analyzed by Fisher exact test.

## Results

**Figure 1:**
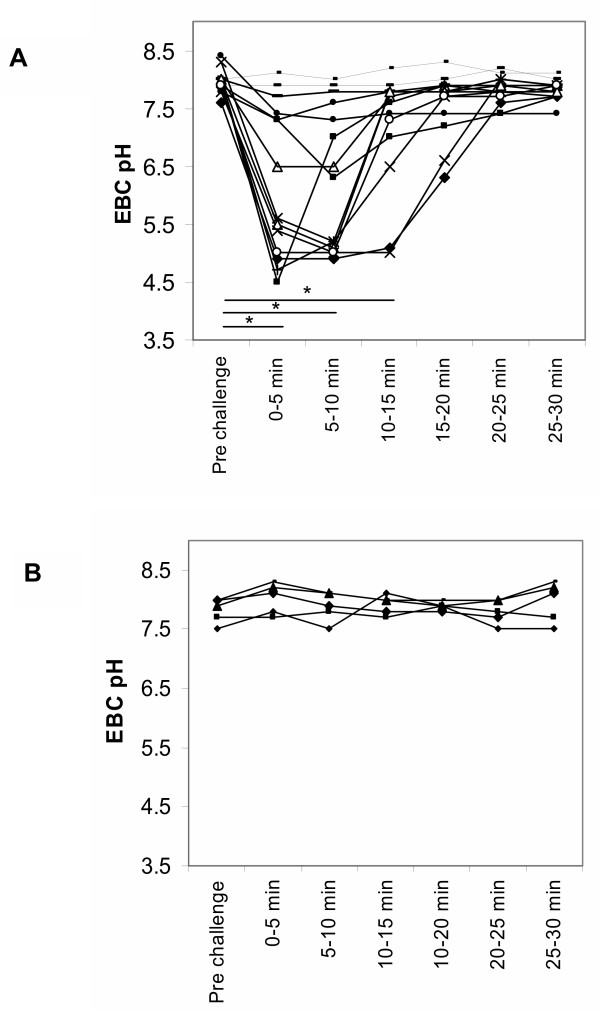
Repeated exhaled breath condensate pH (after gas standardization) before and after pharyngeal challenge by means of an acidic drink (Figure 1A) and after a water control (Figure 1B). EBC pH is transiently low after acid challenge, lasting approximately 10 to 15 minutes. * indicates significant differences from baseline (ANOVA on Ranks with Dunn's test, at p = 0.05).

Ingestion of acidic beverage (as an effort to temporarily acidify the hypopharynx) caused a rapid and pronounced and significant EBC pH decline that persisted for 10–20 minutes (p < 0.05, Figure 1A). This provided the evidence to suggest that EBC sample collection initiated by the patient within 10 minutes of coughing would generally identify if the pharynx (and possibly lower airway) was acidic at the time. Ingestion of water did not affect EBC pH (Figure 1B).

44 subjects were enrolled to provide a full EBC pH profile by collecting all samples over a 1–4 day period in their homes. In healthy subjects (n = 22, age 35 ± 17 years), the median (25–75% range) pH of all EBC samples was 8.1 (8.0–8.2) (n = 174 individual samples) which was essentially identical to the Paget-Brown normative database of 404 individual collections from healthy subjects[6]. In regards to the EBC pH profile, 18/22 subjects revealed an EBC pH profile consisting of entirely normal EBC pH values (defined based on Paget-Brown[6] to be greater than or equal to 7.4). Of the 174 samples collected from controls, 6 samples had a low EBC pH. Two subjects each had 1 low pH value out of their 8 samples, and two subjects had 2 low pH values.

22 chronic cough subjects performed the EBC pH profile (age 28 ± 23 years). These patients were likely to have various causes of their cough. In these subjects, the median EBC pH of all the individual samples collected was 7.9 (7.6–8.0, n = 166), which was only minimally, but statistically significantly, lower than controls (p < 0.001). 29/166 samples revealed a low EBC pH value (p < 0.001 compared to this proportion in controls).

Of the 22 patients prescribed PPI therapy, 17 patients filled the PPI prescription and initiated therapy as directed by the physician. The other 5 patients never filled the prescription, however follow-up information was available from all subjects. EBC pH profiles were compared to responsiveness to PPI therapeutic challenge or to untreated outcome. After 1 month of therapy with PPI, 8 subjects reported a positive response (75% or more improvement in cough symptoms) and 9 subjects were classified as non-responders (0 or 25% improvement). No subject reported a 50% (equivocal) improvement. Of the 5 patients who did not start the PPI, all reported substantial resolution (75–100%) of symptoms spontaneously.

**Figure 2:**
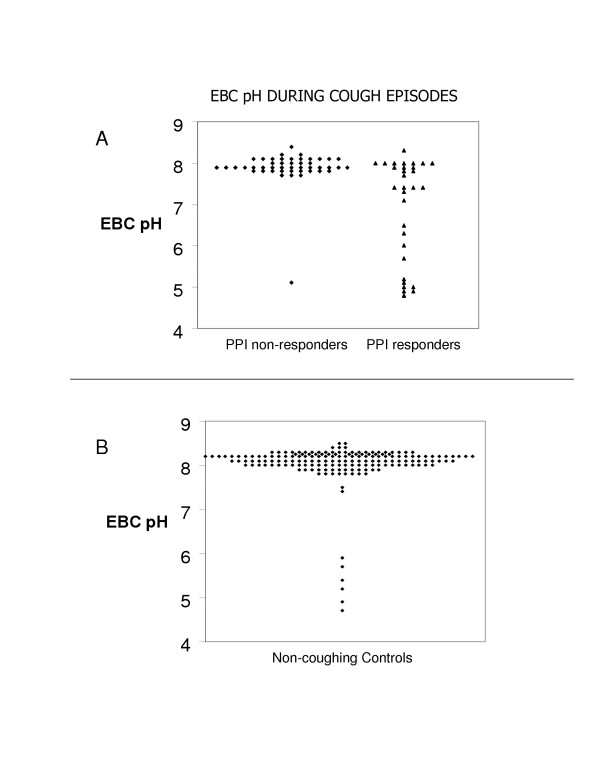
A. Individual isolated exhaled breath condensate pH values immediately after coughing episodes in patients grouped by the response of their cough to a subsequent 1 month trial of proton pump inhibition. There are multiple samples collected from each subject. EBC acidification is significantly more common during cough in patients who subsequently respond to proton pump inhibition. B. Individual EBC pH data points plotted from 22 control subjects, for comparison.

Subjects whose cough responded to PPI therapy were significantly more likely to have one or more of their coughing episodes occurring in the setting of a low EBC pH than those who did not respond to acid blockade (p = 0.001). 14 out of 32 coughing episodes in these 8 PPI responders occurred in association with a low EBC pH value, and an additional 4 had equivocally low pH value (pH = 7.4). Of the coughing episodes in the 9 PPI non-responders, only 1 out of 47 occurred in association with a low EBC pH, and none had equivocal values (equivocal = pH 7.4). These individual assay data points are graphically presented in Figure 2.

One key purpose of this study was to move away from reliance on analysis of single EBC values, and instead to investigate EBC pH *profiles *derived from all samples collected from each individual subject along with the recorded concurrent symptoms. This was possible because of the availability of a kit designed for multiple collections of EBC samples in patients' homes. To demonstrate this multi-sample EBC pH assay system more clearly, a typical positive EBC pH profile from this study is shown in Table 2.

**Figure 3:**
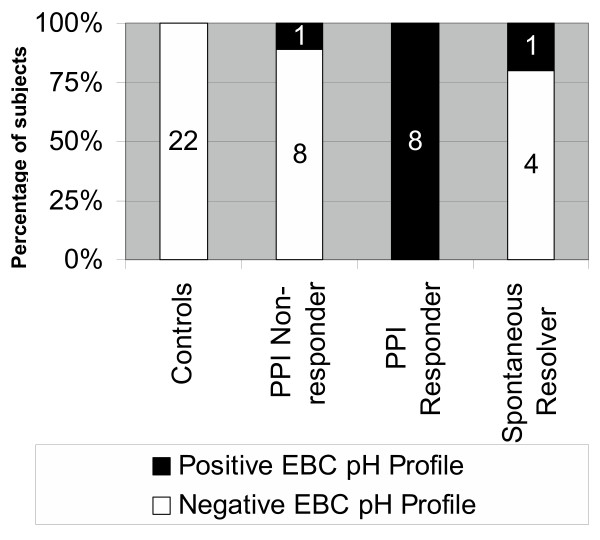
Multi-sample EBC pH profiles of controls and patients. Chronic cough patients are separated into three groups: those who subsequently showed minimal or no response to proton pump inhibition (PPI Non-responders); those who had substantial clinical response to proton pump inhibition (PPI responders), and those who elected to not take the prescribed proton pump inhibitor but who nonetheless had substantial improvement in cough (Spontaneous Resolver). EBC pH profiles are noted as positive if a cough was associated with a low EBC pH value on 1 or more occasions and one or more other EBC pH values was normal. Note the high predictive values of positive and negative EBC pH profiles for response to proton pump inhibition.

Interpreting the data in this context of a multi-sample profile drew a much sharper contrast between PPI responders and non-responders than did the use of individual collections. In this study, the predictive value of a positive EBC pH profile (again, defined as one or more coughs associated with a low EBC pH value with at least one normal EBC pH value at another time), in terms of responsiveness to PPI, was 89%. The predictive value of a negative EBC pH profile was 100% (Figure 3).

Of the 5 subjects who did not begin taking their PPI medication as prescribed, all showed spontaneous resolution of the cough when contacted at one month. 4 of these 5 subjects had a negative EBC pH profile.

Three subjects provided a repeated series of EBC collections in their homes after treatment with PPI and cough resolution. Although few in number, these EBC pH profiles all normalized, with only 1 low EBC pH being found in 24 samples (8 samples each) with that sample having been obtained in the absence of preceding cough).

## Discussion

The EBC pH profile developed for this project is novel methodology that distinguishes this approach from previous investigative efforts in which only individual data points were evaluated.

In this study, the presence of one or more episodes of cough with a concurrent low EBC pH value in any subject's EBC pH profile strongly predicted responsiveness of the cough to proton-pump inhibition. In the absence of any coughing episodes occurring in association with a low EBC pH value, the patient's responsiveness to proton pump inhibition was minimal or non-existent. The patients were recruited into this study based on the intention of their respiratory medicine physician to initiate a trial of proton pump inhibition for suspected acid-reflux cough. Despite being enrolled from a subspecialty clinic, half of such patients did not respond to a one-month trial of twice daily PPI therapy, and these PPI therapeutic failures could be well identified by a multi-sample EBC pH profile revealing a series of coughs associated with normal EBC pH values.

Trials of proton pump inhibition have become common for diagnosis of respiratory symptoms associated with acid reflux[7]. This standard is tarnished by the expense and the long period sometimes necessary for efficacy to be evident (1–3 months in most studies). Spontaneous resolution of various causes of chronic cough certainly occurs, and indeed is revealed in the 5 patients in our study who did not start their prescribed PPI therapy. Spontaneous resolution occurring during a PPI therapeutic trial leads to misdiagnoses, and prolonged courses of unnecessary and expensive medication. PPI therapeutic trials also suffer from confusion in the setting of asthma, chronic obstructive pulmonary disease or other respiratory conditions, which may undergo exacerbation coincidently during a PPI trial, making the PPI trial seem ineffective.

Esophageal pH probes are not sufficiently helpful for diagnosis of acid reflux cough. They are expensive, uncomfortable, and neither particularly sensitive nor specific[8]. In clinical practice, esophageal pH probes are most commonly interpreted by gastroenterologists, using criteria developed for GERD diagnosis, which when considered carefully are fairly irrelevant to acid-reflux induced respiratory disease. What is considered a normal amount of acid reflux by these criteria may be profoundly abnormal if each acid event reaches the larynx and triggers cough. The presence of abnormal GER is very common in obstructive lung disease and it is unwise to be confident that it is causing cough just because it is present. Nasopharyngeal or hypopharyngeal placement of a pH probe sensor is particularly uncomfortable for the patient, and is generally reserved for research use. No effort was made to compare EBC pH with invasively measured pharyngeal pH in this study, although that is being undertaken.

Our findings regarding EBC pH and responsiveness to proton pump inhibition differ from those published recently[9], but then the methodology is also different. The previous study of EBC pH in chronic cough used a large non-portable EBC collection device, and therefore the timing of the sample collection was one of convenience in association with a study clinic visit, and not related to an active cough. We provided disposable, portable EBC collectors for the patients to use in their homes, which allowed for targeting of sample collection to within minutes of a coughing episode. If acid reflux was contributing to their cough through hypopharyngeal/laryngeal/tracheal acidification, the EBC pH effect should be brief. If there is only a 0.1 % hypopharyngeal acid contact time, consisting of multiple brief acid reflux events, there will only be 0.1% of the day when the pH will be low. Although this brief acid exposure may be sufficient to cause frequent cough, it will only be identified if the breath sample is collected when the coughing occurs. Therefore timing is critical. This key element of our methodology, using the symptoms of cough to prompt the patient to perform the EBC collections, allows for relevant acid reflux event to be identified, no matter how infrequent and brief.

A final, and critical, difference is that we collected multiple samples from each subject to develop an EBC pH profile, and this profile allows for much greater sensitivity of the procedure by enhancing the likelihood of finding a correlation of cough with acidity if it is indeed present in a given patient.

Coughing may trigger a pharyngeal reflux event, although studies specifically examining concurrent acid reflux and cough find that it is far more common for the reflux to precede the cough[10]. Although we cannot exclude the possibility that in some patients a low EBC pH may result from reflux secondary to cough, this seems unlikely given our data. In this regard, those patients who subsequently did not respond to proton pump inhibition nonetheless had substantial cough symptoms, but without low EBC pH values.

EBC pH has been reported to be low in acute asthma, stable moderate-to-severe asthma, COPD, and the common cold[1]. We believe it is a mistake to attribute each of these to acid reflux and aspiration. Acid reflux can certainly acidify the airway, but it is just one of several pathways leading to airway acidification. Acids emanating from any level of the airway can contribute to the exhaled acids that determine pH[11,12]. Data from intubated lung-healthy patients reveals values essentially identical to normal orally-breathing controls[5], but patients intubated for respiratory illness have been found to have low EBC pH even when there is a cuffed endotracheal tube in place[13] (which will decrease, although not totally eliminate, aspiration). Acidification occurs with inflammation in most every other fluid in the body; there is no reason to think it should be any different for the airway lining fluid.

Distinguishing acid reflux-induced airway acidification from primary lower airway acidification possibly may be accomplished by seeing normal EBC pH values close in time to low EBC pH values. Reflux leads to rapid airway acidification (at least of the hypopharynx, and in many cases the tracheobronchial tree as well). Rate of neutralization of this acid insult likely varies, but seems to be rapid in general. A persistently low EBC pH value over the course of hours may be more suggestive of an acidification process other than reflux.

Our study would have benefited from a more objective cough score as opposed to a subjective scoring system that has not undergone extensive validation. However, we believe this is overcome in this study because the determination as a PPI responder or a non-responder (in terms of subjective cough score) was in no case equivocal. The number of subjects enrolled was sufficient to identify a highly statistically significant association between a low EBC pH (with cough) and responsiveness of cough to proton pump inhibition.

Although there was a trend for different sex distributions between the control groups and the coughing subjects, there were no statistically significant differences in the sexes (by Fisher Exact Test). Additionally, there were no statistically significant differences in the ages of the groups (by ANOVA). However, as a general comment on the likelihood that a patient will respond to PPI therapy, there was a trend for the PPI responsive group to be younger than the PPI non-responders.

## Conclusion

In conclusion, we have tested the ability of serial (multi-sample) collections of exhaled breath condensate with gas-standardized pH measurement to identify – with high positive and negative predictive values – the likelihood of a patient having a positive response to proton pump inhibition prescribed for their chronic cough. Our data suggest that airway acidification occurs in PPI responders, supporting that hypopharyngeal acidification and probably microaspiration are important contributors to PPI responsive acid reflux cough.

Multi-sample measurements of EBC pH in potential study volunteers may be able to decrease the confounding influence of acid reflux cough in future studies designed to test the efficacy of new therapies aimed at the non-acid components of COPD, asthma, and other respiratory diseases. This method of serial EBC pH testing allows for earlier non-invasive diagnosis of acid reflux as a cause of a patient's cough. It should also help more rapidly and efficiently direct the use of PPI medications to the patients likely to respond. Although not yet studied, it is reasonable to expect that this non-invasive tool will lead to more efficient use of medical resources, for example limiting the need for pH probes. This testing has recently started to be used in clinical practice and represents the maturation of the EBC research technique into a rational clinical diagnostic.

## Abbreviations

EBC Exhaled Breath Condensate

PPI Proton Pump Inhibitor

GER Gastroesophageal Reflux

GERD Gastroesophageal Reflux Disease

## Competing interests

JH and BG are cofounders of Respiratory Research, Inc., a company that manufactures the exhaled breath condensate collection equipment used in this study. They are both inventors and intellectual property holders of EBC pH assay methodology.

## Authors' contributions

JH – first and senior author; YY – planned experiments and data collection, assisted with manuscript preparation; JB – patient recruitment, study design and manuscript preparation; BG – assisted with study design, scientific development, subject recruitment; LN – study design, data collection; DB – clinical research coordinator, patient recruitment, follow up, and interactions with Human Investigation Committee; BW – study design, manuscript assistance, patient enrollment; AS – initial study preparations, assay development, manuscript assistance; SH – patient recruitments, documentation development, manuscript preparation.

**Table 1:** Subject characteristics

Subgroup	Age (years)	Sex	EBC pH Median (25–75% range)
Pharyngeal Acid Challenge (n = 15)	28.8 ± 10	10 F 5 M	8.0 (7.9–8.0) (n = 15 samples from before challenge)
Normal Subjects (n = 22)	35.17	13 F 9 M	8.1 (8.0–8.2) (n = 174 samples)
Chronic Cough Subjects (n = 22)	28 ± 23	7 F 15 M	7.9 (7.6–8.0) (n = 166 samples)

**Table 2:** Positive EBC pH profile in a chronic cough patient who subsequently responded well to proton pump inhibition. Note that the patient provides multiple EBC samples over the course of 1 – 2 days, both immediately after coughing, and in the absence of a recent cough (none in previous 1 hour). There are several cough episodes for which there is a low EBC pH value, while other values are normal (revealing transience of the low EBC pH value in this chronic cough patient)

**Date**	**Time**	**Symptom**	**EBC pH**
12/26/04	1545	Cough	8.3
12/26/04	1745	Cough	**5.0**
12/26/04	1945	Cough	7.9
12/27/04	1015	Cough	**6.0**
12/27/04	1215	Cough	7.7
12/27/04	1730	Well	8.1
12/27/04	1930	Well	7.7
12/27/04	2130	Cough	**5.8**
